# Monte Carlo study of TG‐43 dosimetry parameters of GammaMed Plus high dose rate ^192^Ir brachytherapy source using TOPAS

**DOI:** 10.1002/acm2.13252

**Published:** 2021-05-05

**Authors:** Jianan Wu, Yaoqin Xie, Zhen Ding, Feipeng Li, Luhua Wang

**Affiliations:** ^1^ Institute of Biomedical and Health Engineering Shenzhen Institutes of Advanced Technology Chinese Academy of Sciences Shenzhen 518055 China; ^2^ Department of Radiation Oncology National Cancer Center/National Clinical Research Center for Cancer/Cancer Hospital & Shenzhen Hospital Chinese Academy of Medical Sciences and Peking Union Medical College Shenzhen 518116 China; ^3^ Shenzhen Key Laboratory of Advanced Machine Learning and Application College of Mathematics and Statistics Shenzhen University Shenzhen 518060 China; ^4^ Department of Radiation Oncology National Cancer Center/National Clinical Research Center for Cancer/Cancer Hospital Chinese Academy of Medical Sciences and Peking Union Medical College Beijing 100021 China

## Abstract

**Purpose:**

To develop a simulation model for GammaMed Plus high dose rate ^192^Ir brachytherapy source in TOPAS Monte Carlo software and validate it by calculating the TG‐43 dosimetry parameters and comparing them with published data.

**Methods:**

We built a model for GammaMed Plus high dose rate brachytherapy source in TOPAS. The TG‐43 dosimetry parameters including air‐kerma strength *S*_K_, dose‐rate constant *Λ*, radial dose function *g*_L_(*r*), and 2D anisotropy function *F*(*r*,*θ*) were calculated using Monte Carlo simulation with Geant4 physics models and NNDC ^192^Ir spectrum. Calculations using an old ^192^Ir spectrum were also carried out to evaluate the impact of incident spectrum and cross sections. The results were compared with published data.

**Results:**

For calculations using the NNDC spectrum, the air‐kerma strength per unit source activity *S*_K_/*A* and *Λ* were 1.0139 × 10^‐7^ U/Bq and 1.1101 cGy.h^−1^.U^−1^, which were 3.56% higher and 0.62% lower than the reference values, respectively. The *g*_L_(*r*) agreed with reference values within 1% for radial distances from 2 mm to 20 cm. For radial distances of 1, 3, 5, and 10 cm, the agreements between *F*(*r*,*θ*) from this work and the reference data were within 1.5% for 15° < *θ* < 165°, and within 4% for all *θ* values. The discrepancies were attributed to the updated source spectrum and cross sections. They caused deviations of the *S*_K_/*A* of 2.90% and 0.64%, respectively. As for *g*_L_(*r*), they caused average deviations of −0.22% and 0.48%, respectively. Their impact on *F*(*r*,*θ*) was not quantified for the relatively high statistical uncertainties, but basically they did not result in significant discrepancies.

**Conclusion:**

A model for GammaMed Plus high dose rate ^192^Ir brachytherapy source was developed in TOPAS and validated following TG‐43 protocols, which can be used for future studies. The impact of updated incident spectrum and cross sections on the dosimetry parameters was quantified.

## 1 INTRODUCTION

Brachytherapy, a specific form of radiation therapy, has been widely used to treat patients with cervical cancer, prostate cancer, uterine endometrial cancer, or breast cancer, etc.^1^ Traditionally, in the commercial treatment planning systems (TPS), the dose distribution for brachytherapy source has been computed by modeling all volumes as water based on the American Association of Physicists in Medicine (AAPM) Task Group No. 43 (TG‐43) report.^2^ However, the impact of patient tissue and applicator heterogeneities and finite patient dimensions are ignored in this approach.^3^ Model‐based dose calculation algorithms (MBDCAs) allow for brachytherapy dose calculations in the heterogeneous medium, but they are currently regarded as only supplements to water‐based dose calculation formalism.^3^, ^4^ Monte Carlo (MC) method is considered the “gold standard” for dose calculation in radiation therapy. Precise dose predictions can be achieved using MC method, especially in highly complex and heterogeneous environments such as human tissue.

Except for the general purpose MC codes used in brachytherapy dose calculations such as Geant4^5^ and EGSnrc,^6^, ^7^ several MC dose calculation engines for brachytherapy applications have been developed including gBMC^8^ and RapidBrachyMCTPS^9^ based on Geant4, as well as BrachyDose^10^ and egs_brachy^11^ based on EGSnrc. MC dose calculations have been used as the ground truth in validating novel applications for brachytherapy. Intensity‐modulated brachytherapy (IMBT) methods are commonly validated by MC dose calculations.^12^ Skinner et al.^13^ investigated the use of high‐Z 3D printed applicators in ^192^Ir IMBT using TOPAS MC code. Mao et al.^14^ developed a deep learning‐based rapid dose calculation engine RapidBrachyDL, which was validated using MC dose calculations with RapidBrachyMCTPS, an MC‐based TPS.

MC and experimental methods are both requested by the updated TG‐43 report^15^ (TG‐43U1) for the determination of the TG‐43 dosimetry parameters for brachytherapy sources. The methodological recommendations for MC‐based dosimetry have been expatiated in TG‐43U1 report as well as its supplementary TG‐43U1S1^16^ and TG‐43U1S2^17^ reports. Ballester et al. did the first MC calculation of the dosimetry parameters of GammaMed Plus high dose rate (HDR) ^192^Ir source using Geant3 MC code,^18^ but the incident ^192^Ir spectrum was not indicated. A similar study was carried out by Taylor and Rogers^6^, ^7^ for more types of sources using EGSnrc with an incident ^192^Ir spectrum from Duchemin and Coursol.^19^ A report from the AAPM High Energy Brachytherapy Source Dosimetry (HEBD) Working Group provided consensus dosimetry datasets for various brachytherapy sources based on published MC calculations and experimental measurements.^20^ After the publication of this report, more precise ^192^Ir spectra have been released,^21^, ^22^ which may render variations of the dosimetry parameters. The TOPAS wrapper code^23^ based on Geant4 contains a different set of cross section data than those used in previous studies; thus, the use of TOPAS in brachytherapy requires validation.

In this study, the Varian GammaMed Plus HDR ^192^Ir brachytherapy source was modeled with TOPAS MC code, and the dosimetry parameters including air‐kerma strength, dose‐rate constant, radial dose function, and 2D anisotropy function were investigated following TG‐43 and TG‐43U1 protocols. The results were validated by comparing with previous published works. The impact of the new incident ^192^Ir spectrum and cross section datasets on the dosimetry parameters was evaluated.

## 2 MATERIALS AND METHODS

### 2.A TG‐43 dosimetry formalism for brachytherapy line sources

#### 2.A.1 2D Dose‐rate formalism

This work followed the 2D dose‐calculation formalisms for line sources given in TG‐43U1 protocol.^15^ The two‐dimensional dose‐rate equation is.

(1) $$\dot{D}\left(r,\theta \right)=S_{K}\cdot \Lambda \cdot \frac{G_{L}\left(r,\theta \right)}{G_{L}\left(r_{0},\theta_{0}\right)}\cdot g_{L}\left(r\right)\cdot F\left(r,\theta \right),$$

where *r* is the distance from the center of the active source, and *θ* is the polar angle relative to the source longitudinal axis. *r*_0_ and *θ*_0_ denote the reference distance and angle, and are specified to be 1 cm and 90°, respectively.

The air‐kerma strength *S*_K_, dose‐rate constant *Λ*, radial dose function *g*_L_(*r*), 2D anisotropy function *F*(*r*,*θ*), and geometry function *G*_L_(r,*θ*) as well as their calculation methodologies were defined in TG‐43U1 protocol. A fifth‐order polynomial fit to the *g*_L_(*r*) data is commonly used.

(2) $$g_{L}\left(r\right)=a_{0}+a_{1}r+a_{2}r^{2}+a_{3}r^{3}+a_{4}r^{4}+a_{5}r^{5}.$$

#### 2.A.2 Air‐kerma strength

Air‐kerma strength *S*_K_ is defined as the air‐kerma rate ${\dot{K}}_{\delta }\left(d\right)$ *in vacuo* at distance *d* located on the transverse plane of the source due to photons of energy greater than *δ*, multiplied by *d* ^2^, and has units of cGy.cm^2^.h^−1^ (these unit combinations are also denoted by U),

(3) $$S_{K}={\dot{K}}_{\delta }\left(d\right)\cdot d^{2}.$$

In this work, ${\dot{K}}_{\delta }\left(d\right)$ per initial photon, ${\dot{k}}_{\delta }\left(d\right)$, is calculated using the following equation^24^

(4) $${\dot{k}}_{\delta }\left(d\right)=1.602\times 10^{‐10}\times \sum_{E_{\text{min}}}^{E_{\text{max}}}\phi \left(E_{i}\right)E_{i}\frac{\mu_{\text{en}}}{\rho }\left(E_{i}\right)\Delta E\left(\text{Gy}\;\text{per}\;\text{initial}\;\text{photon}\right),$$

where *E_i_*(MeV) is the midpoint of each energy bin, $\phi \left(E_{i}\right)$ is the photon fluence per initial photon at energy *E_i_*, $\frac{\mu_{\text{en}}}{\rho }\left(E_{i}\right)$ is the mass energy absorption coefficient at energy *E_i_*, and Δ*E* is the bin size. The air‐kerma strength per unit source activity *S*_K_/*A* is then calculated from

(5) $$S_{K}/A=3.6\times 10^{9}\times {\dot{k}}_{\delta }\left(d\right)\cdot d^{2}\times 2.363\left(U/\text{Bq}\right),$$

where 2.363 is the average number of photons emitted from one ^192^Ir decay.^24^

In this study, dry air was used in the calculations of air‐kerma strength.^17^ The photon fluence was calculated in a 10 × 10 × 0.05 cm^3^ voxel located 1 m from the center of the source on the transverse plane, where 0.05 cm was the dimension along the radial axis of the source. A correction factor of 0.22% should be used to account for the variation of the inverse square law over the scoring region.^7^, ^10^ The mass energy absorption coefficient data for dry air were taken from National Institute of Standards and Technology (NIST) database.^25^

### 2.B Varian GammaMed Plus HDR ^192^Ir source

**Fig. 1:**
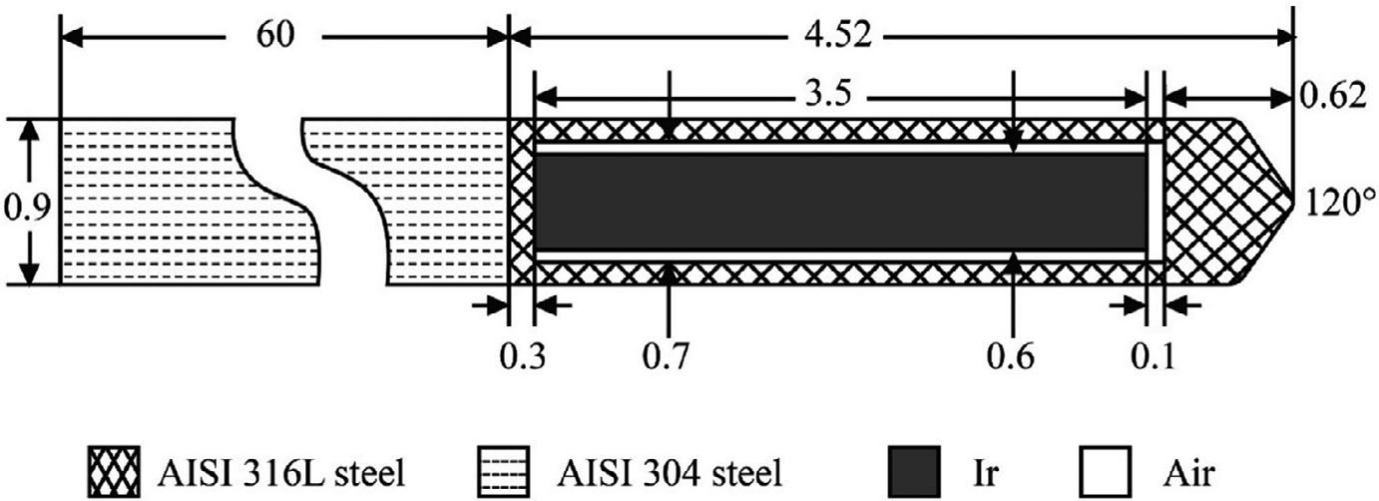
Materials and dimensions (mm) of the Varian GammaMed Plus HDR ^192^Ir source.

The materials and dimensions of the Varian GammaMed HDR Plus source in this simulation were taken from previous studies^7^, ^18^, ^20^ as illustrated in Fig. 1. A 3.5‐mm long Ir core with a diameter of 0.6 mm was enclosed in a 0.9‐mm diameter AISI 316L stainless steel capsule to form the source. A 6‐cm long AISI 304 stainless steel cylinder representing the proximal end of the cable was also included in the simulation. The elements, percentages, and density of each material used in the simulations are shown in Table 1.

**Table 1:** The materials used in Monte Carlo (MC) simulations.

Material	Elements and percentages	Density (g/cm^3^)
Ir	Ir(100)	22.42
AISI 316L steel^a^	C(0.03), N(0.1), Si(0.75), P(0.045), S(0.03), Cr(17), Mn(2), Fe(65.545), Ni(12), Mo(2.5)	7.8
AISI 304 steel^b^	C(0.08), N(0.1), Si(0.75), P(0.045), S(0.03), Cr(19), Mn(2), Fe(67.995), Ni(10)	5.6
Liquid water^c^	H(11.1), O(88.9)	0.998
Dry air^c^	C(0.012), N(75.527), O(23.178), Ar(1.283)	1.197 × 10^−3^

^a^ AK Steel Corporation. *Product datasheet 316/316L stainless steel*. https://www.aksteel.com/sites/default/files/2018‐11/316‐316l‐stainless.pdf. Accessed December 15, 2020.

^b^ AK Steel Corporation. *Product datasheet 304/304L stainless steel*. https://www.aksteel.com/sites/default/files/2018‐11/304‐304l‐stainless.pdf. Accessed December 15, 2020.

^c^ Recommended parameters by TG‐43U1S2 report.^14^

### 2.C Monte Carlo code and the simulation configuration

The TOPAS MC code^23^ is an advanced and user‐friendly extension to Geant4,^26^, ^27^, ^28^ which can be used in simulation studies of various forms of radiotherapy. TOPAS version 3.4 was used in this work with Geant4 version 10.05.p02. The physics modules used in this work were “g4em‐standard_opt4,” “g4h‐phy_QGSP_BIC_HP,” “g4decay,” “g4ion‐binarycascade,” “g4h‐elastic_HP,” and “g4stopping.” The production threshold for all particles was taken as 10 keV following a previous recommendation.^20^ The range cutoff for all particles was taken as 0.05 mm. The maximum step size varied from 0.05 to 1 mm for different voxel sizes.

We used two photon only ^192^Ir spectra from National Nuclear Data Center (NNDC)^21^, ^22^ and Duchemin and Coursol^19^ as the initial source spectra. Volumetric source emitting particles from randomly sampled starting positions from within the Ir core volume were defined. For dose calculations in water, the source was placed at the center of a water cube with dimensions of 80 × 80 × 80 cm^3^. Different voxel sizes ranging from (0.1 mm)^3^ to (2 mm)^3^ were chosen according to the distance from the center of the source to ensure both precision and efficiency. These simulation configurations basically complied to recommendations of TG‐43 reports and other studies.^2^, ^15^, ^16^, ^17^, ^20^ We performed ten runs for each simulation to evaluate the statistical uncertainty. A total of 10^10^ and 10^9^ initial photons were used for each simulation run in air (air‐kerma strength) and in water (dose‐rate constant, radial dose function, and 2D anisotropy function), respectively.

## 3 RESULTS

### 3.A Air‐kerma strength and dose‐rate constant

**Fig. 2:**
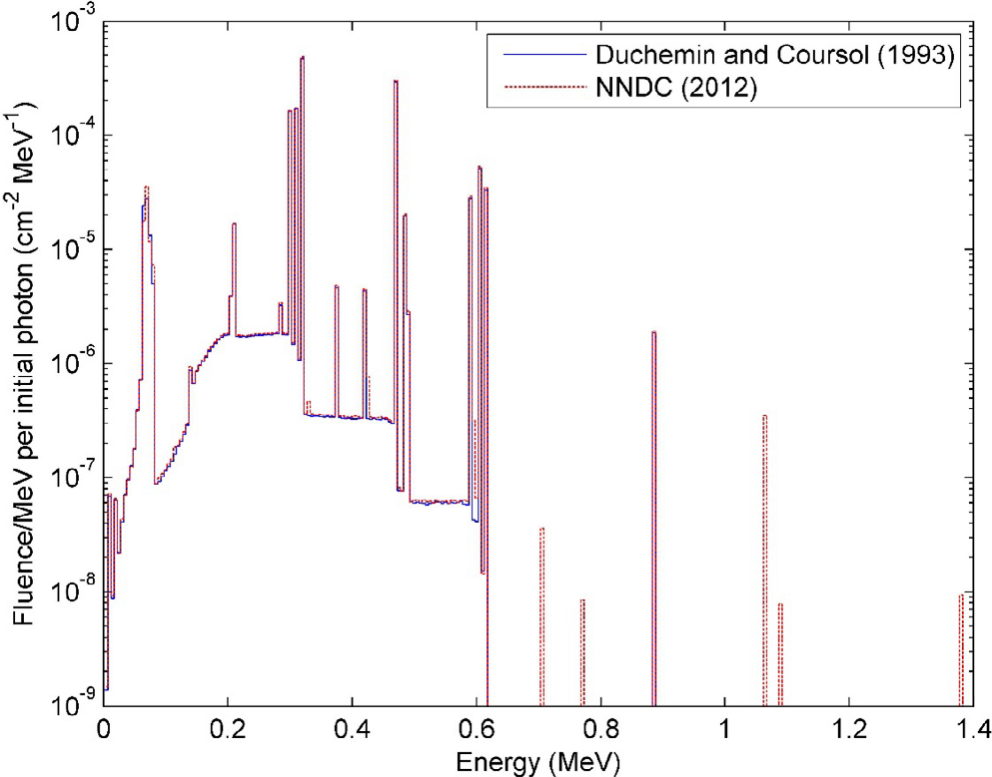
The photon fluence spectrum at 1 m on the transverse plane for the GammaMed Plus HDR ^192^Ir source.

The photon fluence spectrum obtained at 1 m on the transverse plane of the source is reported as fluence per MeV per initial photon in Fig. 2. The bin size is 5 keV. Compared with calculation using Duchemen and Coursol’s spectrum, five extra fluence peaks between 0.7 and 1.4 MeV were observed in the fluence spectrum obtained using NNDC spectrum, and some minor differences under 0.7 MeV were also presented. The calculated air‐kerma strength per unit source activity *S*_K_/*A* is 1.0139 × 10^−7^ U/Bq using NNDC spectrum, and 9.853 × 10^−8^ U/Bq using Duchemen and Coursol’s spectrum, which are 3.56% and 0.64% higher than the reference value^20^ of 9.790 × 10^−8^ U/Bq, respectively. The calculated dose‐rate constant *Λ* is 1.110 cGy.h^−1^.U^−1^ using NNDC spectrum, and 1.106 cGy.h^−1^.U^−1^ using Duchemen and Coursol’s spectrum, which are 0.62% and 0.95% lower than the reference value^20^ of 1.117 cGy h^−1^ U^−1^, respectively. The statistical uncertainties of these parameters were smaller than 0.1%.

### 3.B Radial dose function

**Fig. 3:**
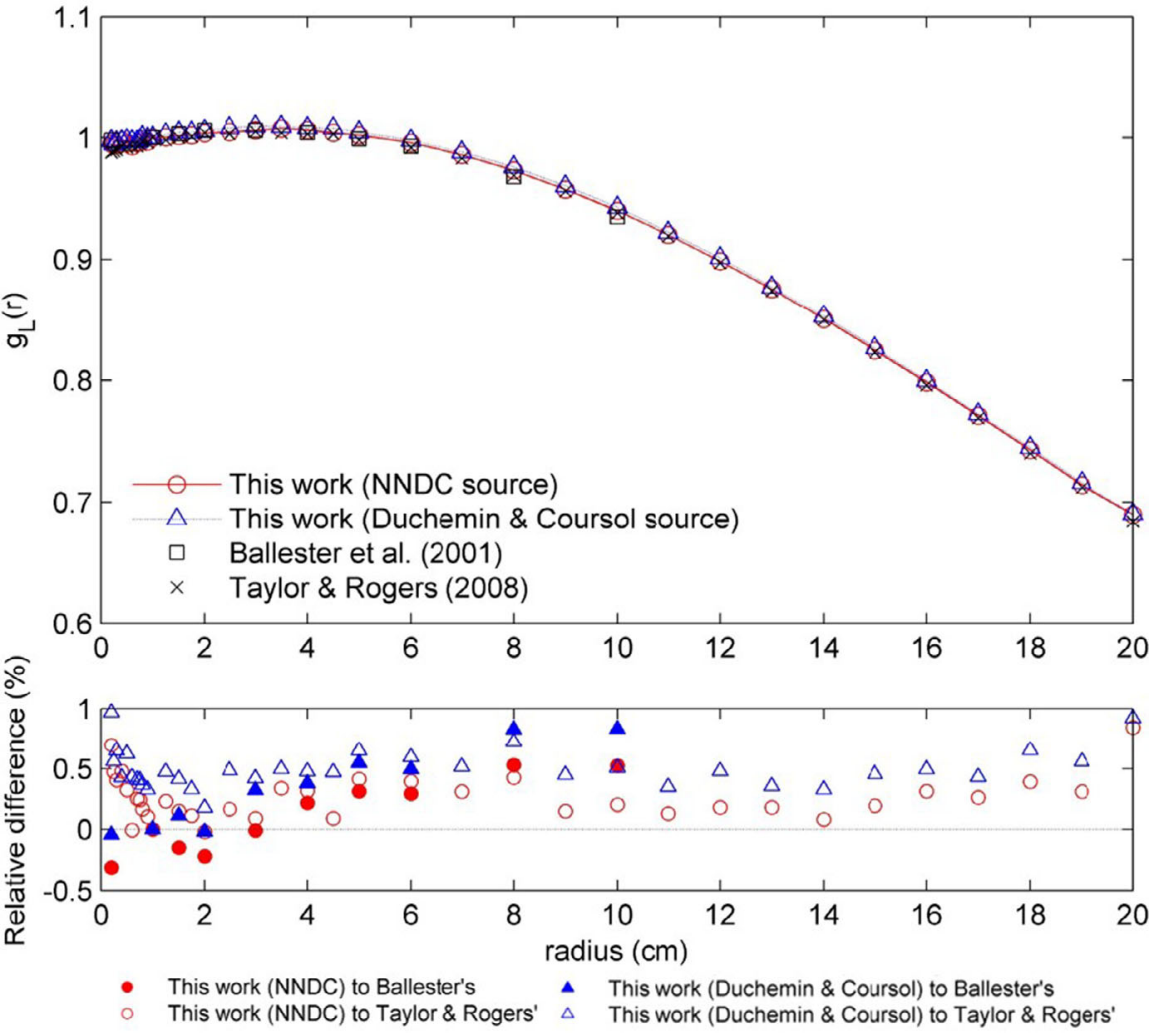
The radial dose function *g*_L_(*r*) for the GammaMed Plus HDR ^192^Ir source. Data from Ballester et al.^18^ and Taylor & Rogers^7^ are also included for comparison. Relative difference (%) to the reference data is indicated.

The radial dose functions *g*_L_(*r*) calculated at radial distances from 2 mm to 20 cm are presented in Fig. 3. Two sets of data from previous works^7^, ^18^ are also shown for comparison reason. The *g*_L_(*r*) calculated using NNDC spectrum was on average 0.22% lower than that using Duchemen and Coursol’s spectrum. Both calculations agreed with both previously published results within 1%. The relative difference of both results to Ballester et al.’s work was negative or small for radius of 2 cm or below, and got larger with the increase of radius. Both calculations were higher than Taylor and Rogers’ work, presenting average deviations of 0.26% and 0.48% for calculations using NNDC and Duchemen and Coursol’s spectrum, respectively. The coefficients *a*_0_ to *a*_5_ in the fitting Eq. (2) were 0.9930, 0.007834, −0.0007279, −0.0001243, 8.184 × 10^−6^, and −1.528 × 10^−7^ for calculation using NNDC spectrum, and 0.9981, 0.00768, −0.0005617, −0.0001419, 8.622 × 10^−6^, and −1.49 × 10^−7^ for calculation using Duchemen and Coursol’s spectrum. The statistical uncertainties of *g*_L_(*r*) were smaller than 0.1%.

### 3.C 2D anisotropy function

**Fig. 4:**
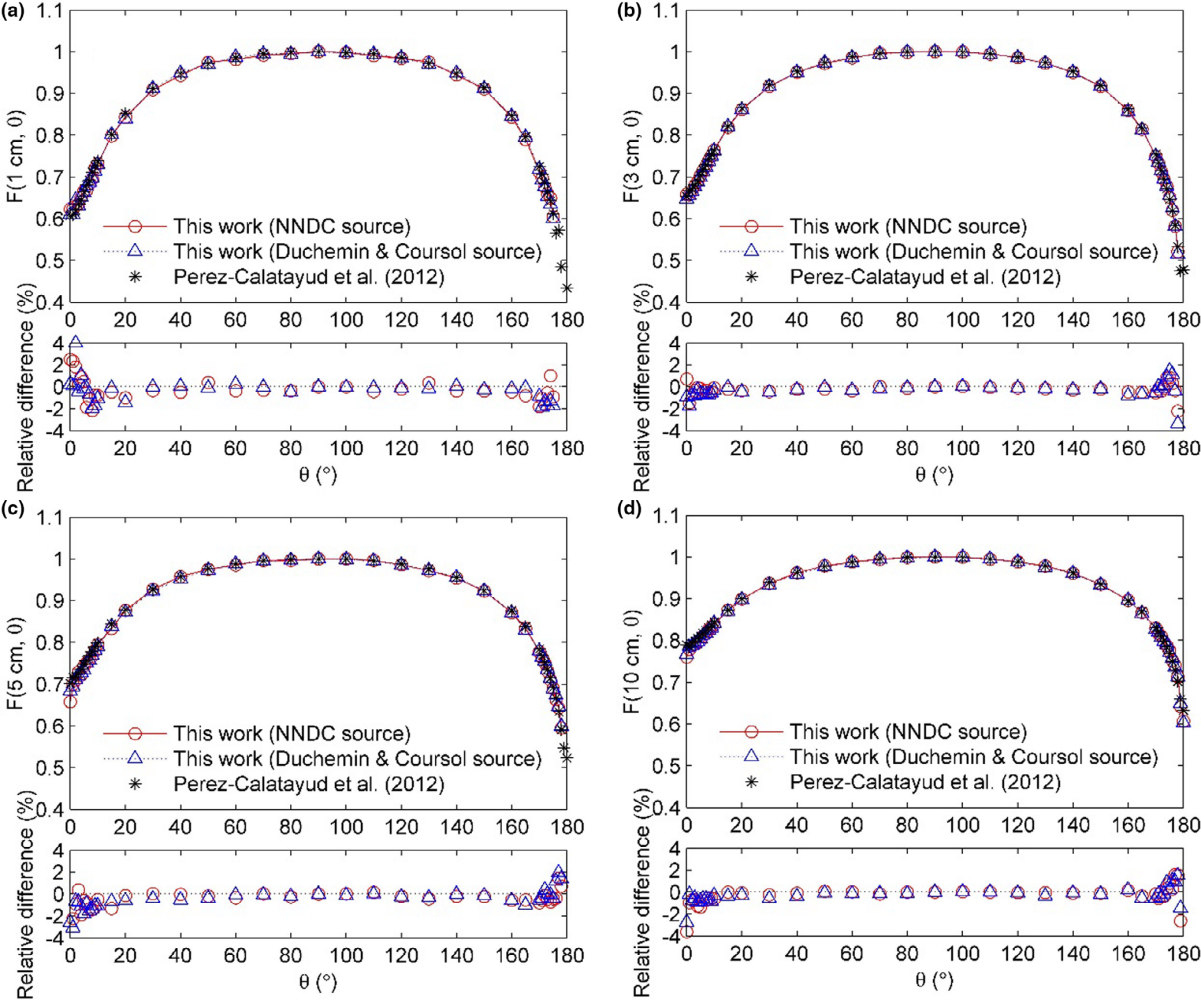
The 2D anisotropy function *F*(*r*,*θ*) for the GammaMed Plus HDR ^192^Ir source for *r* values of (a) 1 cm, (b) 3 cm, (c) 5 cm, and (d) 10 cm. Published data from Perez‐Calatayud et al.^20^ are also indicated for comparison.

Table 2 presents the obtained 2D anisotropy functions *F*(*r*,*θ*) using NNDC spectrum. The 2D anisotropy functions *F*(*r*,*θ*) calculated using two incident spectra at radial distances of 1, 3, 5, and 10 cm are shown in Fig. 4, as well as data from a previous published work.^20^ Calculations inside the source or within 2 mm from the surface of the source are not accurate^7^; thus, these results are not presented. For these radial distances, the agreement between both our calculations and the reference data was within 1.5% for 15°<*θ* < 165°. The relative differences were larger (<4%) for *θ* values closer to 0° and 180°. The average deviations for these radial distances were −0.25%, −0.23%, −0.57%, and −0.38% for calculation using NNDC spectrum, and −0.33%, −0.31%, −0.46%, and −0.34% for calculation using Duchemen and Coursol’s spectrum, respectively. The statistical uncertainties of *F*(*r*,*θ*) were smaller than 1% for 15°<*θ* < 165°, and within 5% for *θ* values closer to 0° and 180°.

**Table 2:** The two‐dimensional (2D) anisotropy function *F*(*r*,*θ*) for the GammaMed Plus high dose rate (HDR) ^192^Ir source.

*θ* (°)	*r*(cm)											
	0.4	0.6	0.8	1	1.5	2	3	4	5	6	8	10
0	0.687	0.666	0.639	0.627	0.618	0.626	0.660	0.674	0.660	0.709	0.756	0.762
1	0.688	0.660	0.635	0.627	0.619	0.628	0.659	0.680	0.694	0.715	0.749	0.780
2	0.691	0.651	0.617	0.628	0.632	0.637	0.666	0.689	0.709	0.725	0.757	0.790
3	0.692	0.646	0.634	0.629	0.645	0.651	0.681	0.705	0.728	0.738	0.771	0.793
4	0.694	0.653	0.645	0.646	0.657	0.666	0.689	0.706	0.726	0.748	0.775	0.796
5	0.697	0.668	0.660	0.672	0.670	0.681	0.702	0.725	0.746	0.755	0.782	0.800
6	0.709	0.683	0.665	0.666	0.684	0.693	0.715	0.738	0.749	0.760	0.792	0.814
7	0.719	0.694	0.693	0.684	0.698	0.705	0.726	0.743	0.760	0.772	0.795	0.818
8	0.726	0.705	0.703	0.695	0.712	0.719	0.739	0.756	0.771	0.787	0.802	0.823
9	0.740	0.720	0.712	0.718	0.726	0.736	0.751	0.766	0.782	0.794	0.815	0.833
10	0.749	0.732	0.727	0.727	0.741	0.748	0.764	0.780	0.796	0.803	0.824	0.840
15	0.811	0.797	0.793	0.798	0.800	0.806	0.818	0.823	0.834	0.847	0.857	0.873
20	0.858	0.847	0.850	0.844	0.849	0.853	0.862	0.868	0.876	0.879	0.889	0.899
30	0.919	0.912	0.906	0.912	0.911	0.913	0.916	0.919	0.928	0.926	0.932	0.936
40	0.949	0.952	0.948	0.941	0.948	0.949	0.952	0.954	0.959	0.955	0.957	0.962
50	0.970	0.973	0.976	0.976	0.971	0.971	0.972	0.974	0.976	0.976	0.975	0.978
60	0.985	0.987	0.987	0.983	0.985	0.985	0.986	0.986	0.984	0.987	0.989	0.988
70	0.987	0.995	0.995	0.992	0.993	0.994	0.997	0.996	0.997	0.995	0.994	0.994
80	0.996	1.001	0.998	0.997	0.998	0.998	1.000	1.000	0.996	0.998	0.998	0.998
90	1	1	1	1	1	1	1	1	1	1	1	1
100	0.996	1.000	0.997	0.995	0.999	0.998	0.999	0.999	1.000	0.998	0.997	0.999
110	0.989	0.997	0.992	0.990	0.995	0.997	0.995	0.993	0.996	0.995	0.993	0.994
120	0.984	0.987	0.986	0.985	0.987	0.985	0.986	0.986	0.986	0.987	0.987	0.988
130	0.969	0.973	0.968	0.978	0.970	0.970	0.972	0.973	0.971	0.973	0.975	0.978
140	0.952	0.952	0.945	0.945	0.946	0.947	0.950	0.951	0.952	0.955	0.959	0.962
150	0.917	0.914	0.911	0.911	0.909	0.914	0.918	0.916	0.923	0.925	0.931	0.933
160	0.857	0.849	0.844	0.845	0.846	0.853	0.860	0.866	0.872	0.876	0.886	0.896
165	0.812	0.799	0.792	0.789	0.797	0.803	0.816	0.823	0.836	0.840	0.856	0.866
170	0.756	0.728	0.720	0.709	0.723	0.732	0.750	0.765	0.780	0.789	0.812	0.830
171	0.747	0.706	0.693	0.701	0.704	0.717	0.733	0.748	0.765	0.777	0.800	0.818
172	0.743	0.689	0.673	0.676	0.684	0.700	0.714	0.735	0.752	0.762	0.789	0.810
173	/	0.667	0.652	0.660	0.665	0.674	0.695	0.712	0.732	0.747	0.780	0.794
174	/	0.658	0.629	0.655	0.640	0.650	0.678	0.698	0.707	0.734	0.758	0.788
175	/	/	0.614	0.609	0.613	0.626	0.652	0.677	0.691	0.716	0.746	0.777
176	/	/	0.597	0.691	0.581	0.593	0.620	0.642	0.665	0.692	0.730	0.751
177	/	/	/	/	0.548	0.547	0.582	0.611	0.643	0.661	0.708	0.740
178	/	/	/	/	/	/	0.521	0.550	0.597	0.609	0.665	0.713
179	/	/	/	/	/	/	/	/	/	/	0.598	0.642
180	/	/	/	/	/	/	/	/	/	/	0.533	0.611

“/” indicates that this position is inside of the source or within 2 mm from the surface of the source, thus the value is not shown.

## 4 DISCUSSION

In this study, the TG‐43 dosimetry parameters for Varian GammaMed Plus HDR ^192^Ir brachytherapy source were calculated using TOPAS MC code. Two different incident ^192^Ir spectra were utilized. The results calculated using both incident spectra were compared with previously published data and showed good agreements, thus validated this implemented MC model.

Monte Carlo simulations and experiments are both requested by TG‐43U1 in determining the TG‐43 dosimetry parameters for brachytherapy sources. Current consensus TG‐43 dataset^20^ for the GammaMed Plus HDR source was based on two MC studies by Ballester et al.^18^ in 2001 and Taylor and Rogers^7^ in 2008. However, new ^192^Ir source spectrum,^21^, ^22^ MC codes, and cross section data for interaction processes were released afterward. MC calculated TG‐43 dosimetry parameters using new configurations were carried out in this study and compared with previously published data, thus validated the proposed MC model and provided a new TG‐43 dataset. Implemented in TOPAS, an easier‐to‐use application of Geant4 MC code for the medical physicist, this model can be easily utilized in further studies regarding MC calculations such as IMBT and microdosimetric studies (along with the TOPAS‐nBio extension).^29^, ^30^

The deviation between calculations could be attributed to different source spectra and cross sections. A previous research^31^ indicated that different ^192^Ir source spectra can cause *S*_k_ differences up to 2%. The relative difference was reported to be as high as 1%^32^ for Compton scattering attenuation coefficient in water between Geant4 “g4em‐standard_opt4” physics model used in our study and XCOM photon cross sections used in Taylor and Rogers’ work. In this work, the *S*_k_/*A* value calculated using new incident spectrum showed 3.56% relative difference from the reference, and that calculated using the old spectrum also used by Taylor and Rogers showed 0.64% difference. This indicated that the majority of the *S*_k_ difference (2.90%) was attributed to different source spectra, while a small portion (0.64%) was caused by different cross sections.

The functions *g*_L_(*r*) and *F*(*r*,*θ*) near the source (*r* ≤ 2 mm) were not accurate thus not displayed because electronic equilibrium may not exist and the dose contribution from the beta spectrum of ^192^Ir average energy of 181 keV is ignored using photon spectrum.^33^, ^34^ The use of new source spectrum caused average decrease of 0.22% for *g*_L_(*r*), which was observed from comparison between our calculations using different source spectra. But by comparing our work and Taylor and Rogers’ both using the old spectrum, we observed that new cross sections seemed to have caused average increase of 0.48% for *g*_L_(*r*).

Less incident photons were simulated in *F*(*r*,*θ*) calculations because they were very time consuming, which caused the relatively high statistical uncertainties. Larger statistical uncertainties of *F*(*r*,*θ*) for *θ* values close to 0° and 180° were due to the small voxel size near the axis of the source. This can be improved in further studies by using more simulation histories. In this case, we conclude that the updated ^192^Ir spectrum and cross sections did not result in significant discrepancies of *F*(*r*,*θ*); however, their impact cannot be quantified in this study.

## 5 CONCLUSION

The Varian GammaMed Plus HDR ^192^Ir brachytherapy source model was implemented within TOPAS MC code. The TG‐43 dosimetry parameters including air‐kerma strength, dose‐rate constant, radial dose function, and 2D anisotropy function were investigated. The calculated results showed good agreements with previous published data. The impact of ^192^Ir spectrum and cross sections used in the simulations on the dosimetry parameters was evaluated. This validated model can be used for further studies involving MC simulations.

## CONFLICT OF INTEREST

No conflicts of interest.

## AUTHOR CONTRIBUTIONS

Jianan Wu conceived the idea, developed the code, analyzed the results, and composed the manuscript. Yaoqin Xie checked results and revised the manuscript. Zhen Ding helped in conceiving the idea and did part of the data analysis. Feipeng Li provided guidance in coding. Luhua Wang revised the manuscript.

## Data Availability

The data that support the findings of this study are available from the corresponding author upon reasonable request.
